# Suppression of the Immune Response by Syngeneic Splenocytes Adoptively Transferred to Sublethally Irradiated Mice

**DOI:** 10.32607/actanaturae.11252

**Published:** 2021

**Authors:** A. A. Kalinina, L. M. Khromykh, D. B. Kazansky, A. V. Deykin, Yu. Yu. Silaeva

**Affiliations:** Federal State Budgetary Institution “N.N. Blokhin National Medical Research Center of Oncology” of the Ministry of Health of the Russian Federation, Moscow, 115478 Russia; Core Facility Centre, Institute of Gene Biology, Russian Academy of Sciences, Moscow, 119334 Russia; Center for Precision Genome Editing and Genetic Technologies for Biomedicine, Institute of Gene Biology, Russian Academy of Sciences, Moscow, 119334 Russia

**Keywords:** memory-like T cell, lymphopenia, homeostatic proliferation, CD44, CD62L

## Abstract

The peripheral T-cell pool consists of several, functionally distinct populations of CD8^+^ T cells. CD44 and CD62L are among the major surface markers that allow us to define T-cell populations. The expression of these molecules depends on the functional status of a T lymphocyte. Under lymphopenic conditions, peripheral T cells undergo homeostatic proliferation and acquire the memory-like surface phenotype CD44hiCD62Lhi. However, the data on the functional activity of these cells remains controversial. In this paper, we analyzed the effects of the adoptive transfer of syngeneic splenocytes on the recovery of CD8^+^ T cells in sublethally irradiated mice. Our data demonstrate that under lymphopenia, donor lymphocytes form a population of memory-like CD8^+^ T cells with the phenotype CD122+CD5+CD49dhiCXCR3+ that shares the phenotypic characteristics of true memory cells and suppressive CD8^+^ T cells. *Ex vivo *experiments showed that after adoptive transfer in irradiated mice, T cells lacked the functions of true effector or memory cells; the allogeneic immune response and immune response to pathogens were greatly suppressed in these mice.

## INTRODUCTION

The peripheral T-cell pool is comprised of several functionally distinct CD8^+^ T-cell populations. The major surface markers of these populations are CD44 and CD62L, whose expression defines the activation phenotype and the migration properties of a T cell. CD62L mediates the interaction between a T lymphocyte and cells of the high endothelium venules, as well as its migration within the lymphoid system. CD44, the receptor for hyaluronic acid in the extracellular matrix, allows T lymphocytes to leave the lymphoid system and migrate to the peripheral tissues [1]. The expression profile of these markers varies depending on the functional state of T lymphocytes. Naive T cells have the surface phenotype CD62LhiCD44lo; CD8 clones activated during the primary immune response lose the CD62L expression and become CD62LloCD44hi. Most CD8 effectors die after completion of their role in the immune response; a small portion of them forms a population of long-living memory T cells capable of maintaining a stable pool in the absence of the specific antigen and accelerated immune response to the specific antigen.

Long-living memory CD8 T cells have the CD44hiCD62Lhi phenotype; however, this does not always correlate with the “antigenic experience” of T cells. Indeed, the peripheral T-cell pool in non-immunized gnotobiotic animals contains virtual memory T cells specific to the model antigen [2, 3]. Under lymphopenia, the peripheral T lymphocytes undergo homeostatic proliferation and acquire the surface phenotype of memory T cells: CD44^+^CD62L^+^ (T_ML_, “memory-like” T cells) [4-7]. The T_ML_ population cannot down-regulate the expression of surface activation molecules and acquire a naive phenotype [8, 9]. Thus, this population is phenotypically similar to true memory T cells.

Our vast pool of experimental data on the functional properties of T^ML^ cells remains controversial. Several studies have shown that adoptive transfer of naive CD8^+^ T cells under lymphopenic conditions leads to the formation of a T-cell population with the functional features of true memory cells [10, 11]. However, the localization of this population and the expression profile of the chemokine receptors on these cells differ from those of true memory cells [12]. The T^ML^ population, with immunosuppressive activity, was reported as well [13]. Moreover, under lymphopenic conditions, T-cell clones with high affinity to self MHC molecules (i.e., autoreactive T cells) proliferate and acquire a memory phenotype [14, 15]. A population of CD8^+^CD44^+^CD122^+^ T cells with suppressive activity was reported in several studies [13, 16, 17, 18].

These data suggest that the surface phenotype of T lymphocytes may not reflect their actual functional status, and that the population in question could be incorrectly assigned to long-living memory CD8^+^ T cells. In this work, we investigated the relationship between the expression of the surface markers CD44 and CD62L and the functional properties of CD8^+^ T cells under lymphopenia. We observed that the adoptive transfer of syngeneic lymphocytes to sublethally irradiated mice suppressed the immune response in the mice, and that the effect could be at least partially mediated by T^ML^ CD8^+^ T cells with the phenotype CD122^+^CD5^+^CD^-^ 49dhiCXCR3^+^ acquired from the donor lymphocytes.

## MATERIALS AND METHODS

**Mice**

C57BL/6 (K_b_I-A_b_D_b_), B10.D2(R101) (K_d_I-A_d_I-E_d_D_b_), FVB (K_q_I-A_q_I-E_q_D_q_), and C57BL/6-TgN(ACTbEGFP) 1Osb (K_b_I-A_b_D_b_) (hereafter referred to as B6.GFP) strains were obtained from the breeding facility of the N.N. Blokhin National Medical Research Center of Oncology of the Ministry of Health of the Russian Federation (N.N. Blokhin NMRCO, Moscow, Russia). All the experimental procedures were approved by the Ethics Committee on Animal Experimentation of N.N. Blokhin NMRCO and of the Institute of Gene Biology of the Russian Academy of Sciences (Moscow, Russia).

**Cell lines**

The EL4 lymphoma cells were obtained from the collection of N.N. Blokhin NMRCO. The EL4 cells were transplanted intraperitoneally (i.p.) into syngeneic C57BL/6 mice (3.0–5.0 × 10^6^ cells/mouse) and grown as ascites for 10–14 days. Tumor cells were aseptically aspirated from the peritoneal ascites and washed three times by centrifugation (200 *g*) in a phosphate buffered saline (PBS, pH 7.4) at 4°C. Viable cells were counted after trypan blue/eosin staining in a Goryaev chamber and used for mouse immunization.

**Bacterial strains and growth conditions**

The *Salmonella typhimurium *virulent strain IE 147 and *Listeria monocytogenes *virulent strain EGD were received from the collection of N.F. Gamaleya National Research Center of Epidemiology and Microbiology, the Ministry of Health of the Russian Federation (N.F. Gamaleya NRCEM, Moscow, Russia). The* S. typhimurium *strain was grown overnight in an LB broth (Amresco, USA) at 37°C; tenfold serial dilutions of the culture were then seeded on SS agar (Condalab, Spain), and the colony numbers were counted as described elsewhere. The *L. monocytogenes *strain was grown overnight in BHI broth (BD, San Jose, CA) at 37°C with stirring at 185 rpm on a thermostatic shaker (Shaker-thermostat ES 20 Biosan, Latvia). The resulting culture was diluted 1 : 100 in 200 mL of BHI broth and incubated in a thermostatic shaker at 185 rpm at 37°C until the culture reached an optical density (OD 600) equal to 1.5–1.8. Bacterial titer (CFU/mL) was measured on an ULTROSPEC 10 spectrophotometer (General Electric, USA). Freshly grown cultures of* S. typhimurium *and *L. monocytogenes *were heat-inactivated (1 hr, 60°C; and 90 min, 74°C, respectively) and used in *in vitro *studies.

**Immunization**

B10.D2(R101) mice were immunized i.p. with 2.0 × 10^7^ EL4 cells/mouse. Control non-immunized mice were injected with PBS. After 60 days, mice were euthanized by cervical dislocation; spleens were isolated, and cell suspensions were prepared (see below).

**Irradiation of mice**

Female B10.D2(R101) and C57BL/6 mice were sublethally irradiated (4.5 Gy; Agat-R therapeutic device, Russia; a Co^60^ source with an initial power of 1.9 × 10^14^ Bq). Mice were sacrificed on day 10 post-irradiation, and their splenocytes were used for flow cytometry analyses and *ex vivo *functional tests.

**Cell suspensions**

Splenocytes were homogenized in a Potter homogenizer with a conic pestle in PBS at 4°C and pelleted (200 *g*, 5 min). Red blood cells were lyzed in a lysing buffer (BD Pharmingen, USA). Mononuclear cells were washed three times by centrifugation in PBS at 4°C. The cells were re-suspended in PBS for staining with monoclonal antibodies and adoptive transfer or in the complete medium for *in vitro *tests.

**Adoptive transfer**

**Fig. 1:**
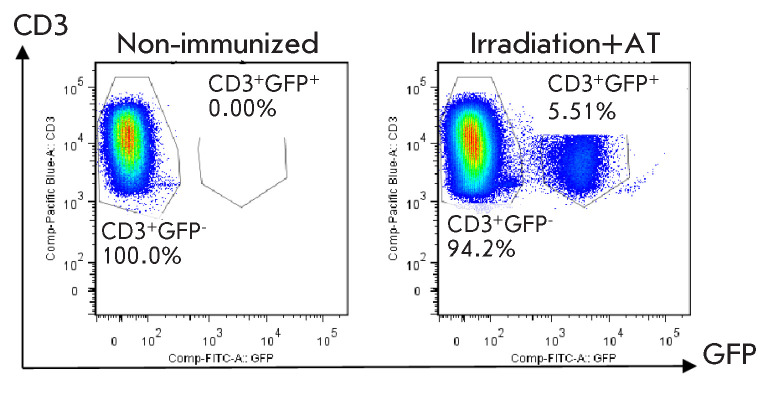
The relative count of GFPhi CD3+ donor cells (GFP+) in the spleen of C57BL/6 mice on day 10 after the sublethal irradiation and adoptive transfer. The data of one representative experiment are shown for 2.5 × 10^6^ events. The data were obtained in three independent experiments, 3 mice per group

Non-immunized B10.D2(R101) mice were irradiated with 4.5 Gy. 24 h post-irradiation; mice were injected i.v. with 1.5 × 10^7^ splenocytes from non-immunized or immunized syngeneic animals. Control irradiated mice received PBS as a placebo in parallel. On day 10 after the adoptive transfer, the splenocytes of the recipient mice were used as responders in *in vitro *tests. Non-immunized C57BL/6 mice were similarly irradiated and injected with the splenocytes of non-immunized B6.GFP mice. On day 10 after the adoptive transfer, the splenocytes of the recipient mice were used for flow cytometry analyses. On day 10 after the adoptive transfer, approximately 5% of GFP^+^ cells were detected in the spleen of irradiated mice (*Fig. 1*).

**Mixed lymphocyte reaction (MLR)**

The spleen cells of FVB ( KqI-AqI-EqDq) and C57BL/6 (KbI-AbDb) mice were used as non-specific and specific stimulators, respectively. The spleen cells of B10.D2(R101) mice were used as the syngeneic control. Stimulator splenocytes were treated with mitomycin C (Kyowa Hakko Kogyo Co., Ltd., Japan) (25 μg/mL, 37°C, 30 min) and washed three times in PBS by centrifugation (200*g*, 5 min, 4°C). Responders (3.0 × 10^5^ cells/well) and stimulators (5.0 × 10^5^ cells/well) were plated (3 : 5) in 96-well U-bottom plates (Corning Costar, Sigma Aldrich, USA) and cultured in 200 μL of a RPMI-1640 medium (PanEco, Russia) supplemented with 10% fetal bovine serum (HyClone, GE Healthcare, USA), 0.01 mg/ mL ciprofloxacin (KRKA, Slovenia), 0.01 M HEPES (PanEco), and 10 mM 2-mercaptoethanol (Merck, Germany) at 37°C with 5% CO_2_, for 72 h. Cell proliferation was measured by incorporation of 3H- thymidine (Isotop, Russia), added in the last 8 h of culturing. The level of cell proliferative activity was expressed as the number of counts per minute (cpm).

***Ex vivo *immune response to pathogens**

5.0 × 10^5^ spleen cells of irradiated B10.D2(R101) mice and irradiated B10.D2(R101) mice 10 days after the adoptive transfer of syngeneic splenocytes from non-immunized mice were plated in 96-well U-bottom plates (Corning Costar, Sigma Aldrich) with 106–107 CFU of heat-inactivated* L. monocytogenes *(strain EGD) or 105 CFU of heat-inactivated *S. typhimurium *(strain IE 147), prepared as described above. The cells were cultured in 200 μL of a RPMI-1640 medium (PanEco, Russia) supplemented as described above at 37°C, with 5% CO_2_, for 72 h. Cells cultured without pathogens were used to assess background proliferation. Cell proliferation was determined as described above. The index of pathogen- induced proliferation was calculated as the ratio between the levels of cell proliferation in response to bacteria and background proliferation.

**Evaluation of EL-4 tumor growth and rejection *in vivo***

Sublethally irradiated B10.D2(R101) mice (with or without adoptive transfer of syngeneic splenocytes) were subcutaneously injected with 0.25 mL of a EL-4 lymphoma cell suspension (8.0 × 10^7^ cells/mL). Tumor nodes were measured on days 7, 14, and 21 post-transplantation. EL-4 lymphoma was considered totally rejected when no subcutaneous tumor nodes were detected at palpation.

**Antibodies**

In this work, the following antibodies were used: anti-CD8α – Percp-Cy5.5 (clone 53–6.7, BD Bioscience, USA), anti-CD62L – APC-Cy7 (clone MEL-14, eBioscience, USA), anti-CD44 – APC (clone IM7, eBioscience), anti-CD3 – PE-Cy7 (clone 145-2C11, eBioscience), anti-CD122 – PE (clone TM-β1, BD Bioscience), anti-CD5- BV421 (clone 53-7.3, BD Biosciences), anti-CXCR3 – BV421 (clone CXCR3-173, BD Biosciences), and anti-CD49d – PE (clone R1-2, BD Biosciences).

**Flow cytometry**

Cell samples (3.0 × 10^6^) were pre-incubated with Fc block (clone 2.4G2, BD Pharmingen, USA) (10 min, 4°C) and then stained with fluorescent antibodies (40 min, 4°C). The analysis was performed on a BD FACSCanto II flow cytometer (BD Bioscience) using the FACSDiva 6.0 software (BD Bioscience). Dead cells were excluded from the analysis based on the parameters of forward and side scatter and staining with propidium iodide (BD Bioscience) or 7-AAD (BioLegend, USA). At least 106 events/samples were collected to characterize the peripheral T-lymphocyte populations. Data were processed using the Flow Jo 7.6 software (TreeStar Inc., USA).

**Statistical analysis**

Data are presented as mean ± SEM. All statistical analyses were performed using the unpaired Student’s t-test. *P-*values < 0.05 were considered significant.

## RESULTS

**Adoptive transfer of syngeneic splenocytes suppresses the immune response in sublethally irradiated mice**

**Fig. 2:**
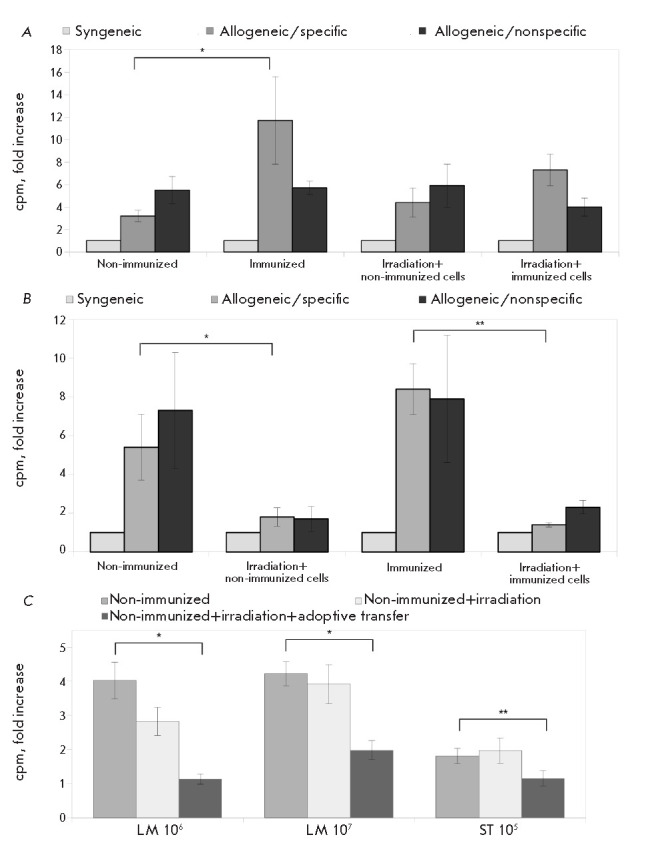
Analyses of the *ex vivo *functional activity of splenocytes in the lymphopenic mice. (*A*) – The relative level of proliferation of the mixed lymphocyte culture of splenocytes of the sublethally irradiated mice in the allogeneic response. The spleen cells of sublethally irradiated mice were used as responders. Mitomycin C-treated splenocytes of syngeneic (B10.D2(R101), allogeneic/specific (C57BL/10), and allogeneic/nonspecific (FVB) mice were used as stimulators. The relative proliferation level was evaluated as a ratio between the allogeneic and syngeneic responses. The data were obtained in three independent experiments, 3 mice per group. (*B*) – The relative level of proliferation of the mixed lymphocyte culture of the splenocytes of sublethally irradiated mice after the adoptive transfer in the allogeneic response. The spleen cells of sublethally irradiated mice on day 10 after the adoptive transfer were used as responders. Mitomycin C-treated splenocytes of syngeneic (B10.D2(R101), allogeneic/specific (C57BL/10), and allogeneic/nonspecific (FVB) mice were used as stimulators. The relative proliferation level was evaluated as a ratio between the allogeneic and syngeneic response. The data were obtained in three independent experiments, 3 mice per group (*p ≤ 0.05; **p ≤ 0.01). (*C*) – The relative level of *in vitro *proliferation of the splenocytes of sublethally irradiated mice after the adoptive transfer in response to pathogens. The splenocytes of sublethally irradiated mice after the adoptive transfer were cultured with 106 CFU/mL (LM 106) or 107 CFU/mL (LM 107) of heat-inactivated *Listeria monocytogenes *or with 105 CFU/mL (ST 105) of heat-inactivated *Salmonella typhimurium*. To assess the background proliferation, the splenocytes were similarly cultured without the pathogen. The relative proliferation levels were assessed as the ratio between the pathogen- induced proliferation and the background proliferation. The data were obtained in three independent experiments, 3–6 mice per group (*p ≤ 0.05, **p ≤ 0.01).

**Fig. 3:**
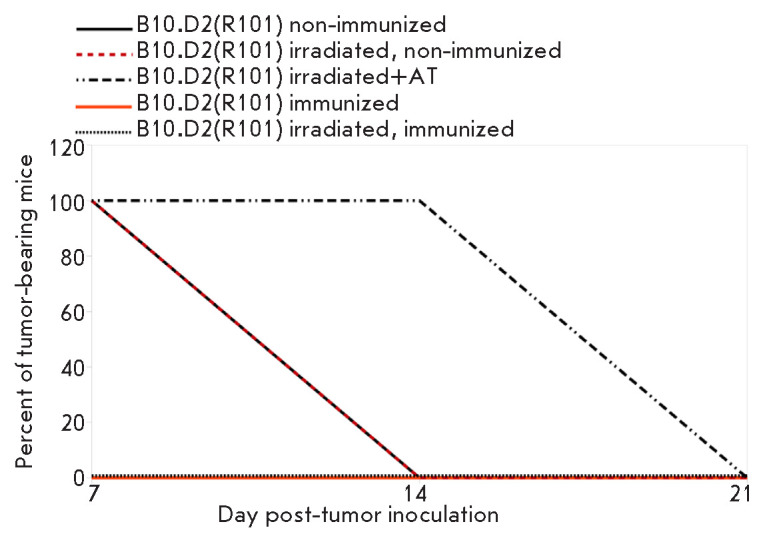
The dynamics of lymphoma EL4 rejection in sublethally irradiated B10.D2(R101) mice after the adoptive transfer. The data of one representative experiment are presented, 3 mice per group

In order to assess the effects of the adoptive transfer of syngeneic splenocytes on the functional status of the immune system in sublethally irradiated mice, we used non-immunized or immunized mice as donors of splenocytes (*Fig. 2A,B*). Irradiation of immunized mice resulted in insignificant (1.6-fold) suppression of the specific immune response compared to the control group of immunized non-irradiated animals, whereas the level of the non-specific immune response remained unchanged (*Fig. 2A*). Dramatic suppression of both specific and non-specific *ex vivo *allogeneic immune responses was observed in irradiated mice with adoptively transferred spleen cells of non-immunized or immunized mice (*Fig. 2B*). Accordingly, irradiated mice after the adoptive transfer exhibited prolonged dynamics of EL-4 lymphoma rejection *in vivo *compared to all control groups (*Fig. 3*).

Moreover, our data showed a significant inhibition of the immune response to both *L. monocytogenes *and* S. typhimurium *in sublethally irradiated mice with the adoptive transfer compared to the control group of irradiated animals (*Fig. 2C*). Notably, the *ex vivo *proliferative response of the splenocytes of irradiated mice without the adoptive transfer remained unchanged compared to the non-irradiated animals (*Fig. 2A,C*).

**Phenotype characteristics of donor and recipient CD3+CD8^+^ T cells in sublethally irradiated mice after an adoptive transfer**

We assumed that the immune response in sublethally irradiated mice after the adoptive transfer of syngeneic splenocytes could be inhibited due to the decrease in the absolute cell count and the relative number of CD3^+^ T cells in the spleen of these mice. To prove this hypothesis true, we performed an adoptive transfer of the spleen cells of B6.GFP mice to sublethally irradiated C57BL/6 mice and individually analyzed populations of the recipient (GFP^-^) and donor (GFP^+^) T cells. Some 5% of GFP^+^ donor cells were detected in the spleen of the irradiated recipients (*Fig. 1*).

**Fig. 4:**
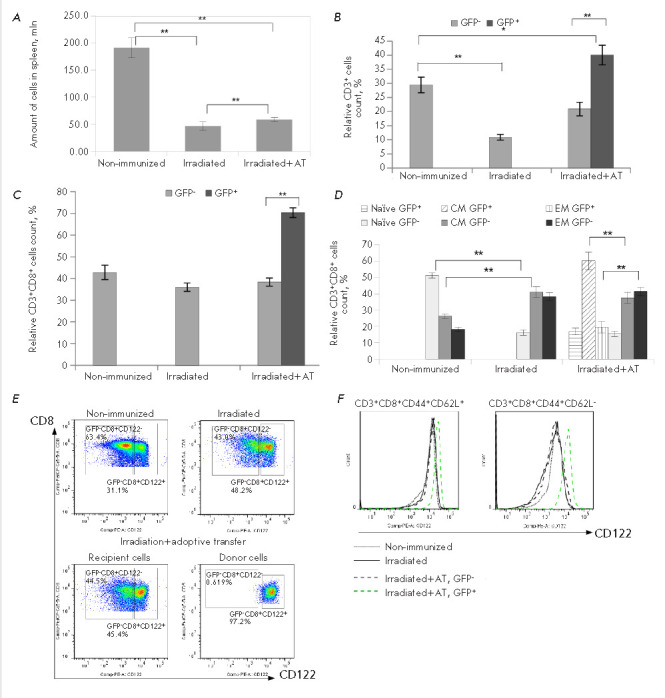
The absolute cell count and the expression profile of activation markers in the population of CD8^+^ T cells of the donor (GFP+) and the recipient (GFP-) sublethally irradiated mice on day 10 after the adoptive transfer. (*A*) – The absolute cell count in the spleen of irradiated mice. The data were obtained in three independent experiments, 6–9 mice per group (**p ≤ 0.01). (*B*) **– **The relative count of CD3+ cells in the spleen of the irradiated mice. The data were obtained in three independent experiments, 4–6 mice per group (*p ≤ 0.05, **p ≤ 0.01). (*C*) **– **The relative count of CD3+CD8^+^ cells in the spleen of the irradiated mice. The data were obtained in three independent experiments, 4–6 mice per group (**p ≤ 0.01). (*D*) – The relative count of CD8^+^ T cells with the phenotype of naive, effectors, and central memory cells. The data were obtained in three independent experiments, 6 mice per group (**p ≤ 0.01). (*E*) – The relative count of CD8^+^CD122+ splenocytes in the population of the donor (GFP+) and the recipient (GFP-) cells of mice on day 10 after the irradiation and adoptive transfer. The data were obtained in three independent experiments, 6 mice per group. The data of one representative experiment are presented. (*F*) – The expression profile of CD122 in the population of CD8^+^CD44+CD62L+ and CD8^+^CD44+CD62L- T cells in the spleen of the mice on day 10 after the irradiation and adoptive transfer. The expression profiles for the donor (GFP+) and the recipient (GFP-) cells. The data were obtained in three independent experiments, 6 mice per group. The data of one representative experiment are presented

The absolute cell counts in the spleen of the irradiated mice were 4.9-fold reduced compared to that in the non-irradiated animals (*Fig. 4A*). The adoptive transfer of syngeneic splenocytes resulted in a 1.5-fold increase in spleen cell counts compared to that in the control irradiated mice (*p *≤ 0.01; *Fig. 4A*).

Sublethal irradiation reduced the relative count of CD3+ cells in the spleen of the mice compared to that in the non-irradiated controls (*Fig. 4B*). On day 10 after the adoptive transfer, the relative count of GFP- CD3+ cells in the spleen of the irradiated mice was approximately equal to the CD3+ cell count in the spleen of the non-irradiated mice (*Fig. 4B*). The relative count of CD3+ donor cells (GFP+) was 2.0-fold higher compared to the relative count of GFP- recipient T cells in the spleen of the irradiated mice after the adoptive transfer (*Fig. 4B*).

The population of CD8^+^ T cells remained unchanged in the spleen of the irradiated mice and the subset of the recipient (GFP^-^) T cells of irradiated mice after the adoptive transfer compared to non-irradiated mice (*Fig. 4C*). However, CD8^+^ cells comprised 70% of the donor (GFP^+^) T lymphocytes in the spleen of the irradiated mice after the adoptive transfer, equal to 1.8 times the relative count of recipient CD3^+^CD8^+^ cells (*Fig. 4C*). We assumed that donor CD8^+^ T cells preferentially survive after the adoptive transfer and undergo homeostatic proliferation in the irradiated host. These data correlate with recent studies indicating that CD8^+^ cells require fewer stimuli for homeostatic proliferation compared to CD4^+^ T lymphocytes [19].

Sublethal irradiation resulted in a decrease in the relative count of naive cells and a 1.8- and 2.3-fold increase in the relative count of central memory cells and effector memory cells, respectively, within the recipient (GFP^-^) CD8^+^ T cells as compared to the non-irradiated mice (*Fig. 4D*). A total of 60% of the donor (GFP^+^) CD8^+^ T cells in the spleen of the irradiated mice after the adoptive transfer had the phenotype of memory cells (*Fig. 4D*).

Several studies have revealed CD8^+^CD122^+^ T cells with suppressive functions [15]. We evaluated the expression of CD122 on the recipient (GFP^-^) and the donor (GFP^+^) CD8^+^ T cells in the spleen of the irradiated mice after the adoptive transfer (*Fig. 4E,F*). Over 97% of the donor (GFP^+^) CD8^+^ T cells acquired the phenotype CD8^+^CD122^+^ (*Fig. 4E*), whereas the relative count of CD8^+^CD122^+^ T cells within the population of the recipient lymphocytes remained unchanged compared to the irradiated and non-irradiated mice (*Fig. 4E*). The level of CD122 expression in the subsets of memory cells (CD44^+^CD62L^+^) and effectors (CD44^+^CD62L^-^) within the donor (GFP^+^) T cells was significantly increased compared to the respective subpopulations of the recipient (GFP^-^) lymphocytes (*Fig. 4F*).

**Fig. 5:**
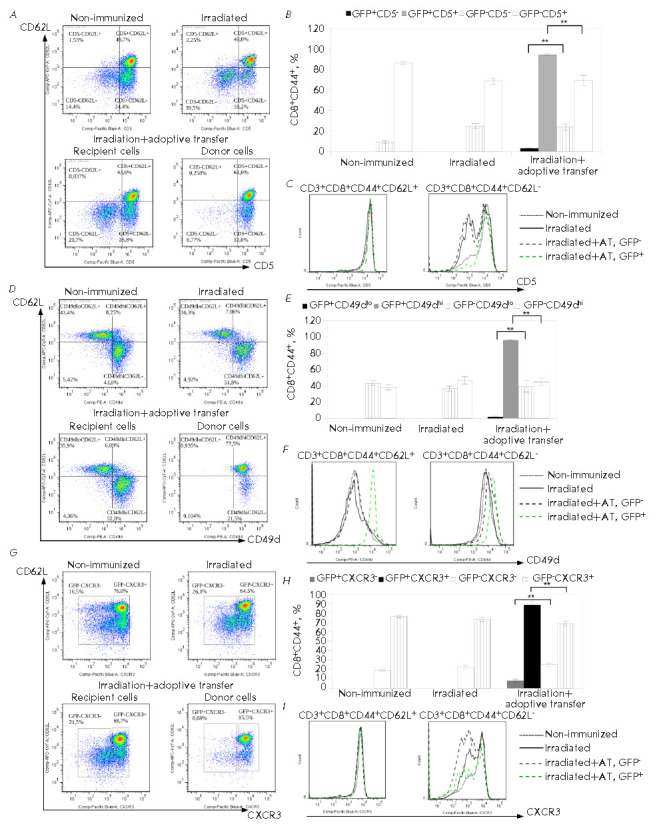
The relative cell count and the expression profile of CD49d, CD5, and CXCR3 in the population CD8^+^CD44+ T cells in the spleen of mice on day 10 after the irradiation and adoptive transfer. Expression of CD5 (*A*), CD49d (*D*), and CXCR3 (*G*) on CD8^+^CD44+ T cells of the donor (GFP+) and the recipient (GFP-) in the spleen of irradiated mice. The data were obtained in three independent experiments, 4–6 mice per group. The data of one representative experiment are presented. The relative count of CD8^+^CD44+ T cells with the phenotypes CD5low and CD5hi (*B*); CD49d- and CD49d+ (*E*); CXCR3- and CXCR3+ (*H*) in the population of the donor (GFP+) and the recipient (GFP-) splenocytes in the irradiated mice. The data were obtained in three independent experiments, 4–6 mice per group. The data of one representative experiment are presented (**p ≤ 0.01). The expression profiles of CD5 (*C*), CD49d (*F*), and CXCR3 (*I*) on CD8^+^CD44+CD62L+ and CD8^+^CD44+CD62L- T cells in the spleen of the irradiated mice. The expression profiles for the donor (GFP+) and the recipient (GFP-) cells are presented. The data were obtained in three independent experiments, 4–6 mice per group. The data of one representative experiment are presented

To evaluate potentially autoreactive T cells within the donor T lymphocytes, we analyzed the expression of CD5 in the population of CD8^+^CD44^+^ T cells (*Fig. 5A,B,C*). Virtually all GFP^+^CD8^+^CD44^+^ T cells expressed CD5 (*Fig. 5A*), while the CD5^+^/CD5^-^ ratio in the population of the recipient (GFP^-^) CD8^+^CD44^+^ cells remained unchanged compared to the control irradiated and non-irradiated mice (*Fig. 5A,B*). The expression level of CD5 in the CD44^+^CD62L^+^ cells was comparable in all experimental groups (*Fig. 5C*).

Some studies have shown suppressive functions for CD8^+^CD122^+^CD49dlow T cells [18]. We evaluated the expression of the CD49d marker in the population of CD8^+^CD44^+^ T cells of the recipient (GFP^-^) and donor (GFP^+^) lymphocytes in the spleen of the irradiated mice after the adoptive transfer (*Fig. 5D,E,F*). Nearly 100% of the donor CD8^+^CD44^+^ T cells acquired the CD49dhi phenotype (*Fig. 5D,E*), whereas the CD49dlow/ CD49dhi ratio in the CD8^+^CD44^+^ T-cell population of the recipient (GFP^-^) cells was similar to that in the irradiated and non-irradiated mice (*Fig. 5D,E*). We observed a significant increase in the CD49d expression level within the CD44^+^CD62L^+^ subset of donor GFP^+^ CD8^+^ T cells (*Fig. 5F*).

Furthermore, over 85% of the donor CD8^+^CD44^+^ T cells expressed a CXCR3 + phenotype (*Fig. 5G,H*). The expression level of CXCR3 in the subpopulation of CD44^+^CD62L^+^ was comparable in all experimental groups; in the subpopulation of donor CD44^+^CD62LT cells, it was in correlation with the level of non-irradiated animals (*Fig. 5I*).

Therefore, the adoptive transfer of syngeneic splenocytes to the lymphopenic host resulted in preferential homeostatic proliferation of CD8^+^ donor T cells that predominantly acquire the phenotype of the central memory cells CD44^+^CD62L^+^, and most donor CD44^+^ T cells carry the CD122^+^CD5^+^CD49dhiCXCR3^+^ phenotype.

## DISCUSSION

Recent data indicate that there is no strict correlation between the surface phenotype and functional characteristics of a memory T cell (long-term self-maintenance, resistance to apoptosis, simplified activation conditions, enhanced proliferation and acquisition of effector functions in response to the specific antigen). The population of CD8^+^CD44^+^CD62L^+^CD122^+^ cells was shown to exhibit immunosuppressive activity [13, 16, 17, 18, 20]. Commonly, this population expresses high levels of the chemokine receptor CXCR3 [17] and low levels of CD49d (CD8^+^CD122^+^CD49dlow) [18]. Similar populations of such suppressive CD8^+^ T cells were detected both in mice and in humans [21].

We have shown that the adoptive transfer of syngeneic lymphocytes to irradiated mice results in the suppression of the allogeneic immune response and the immune responses to pathogens in such mice. This could be explained by the preferential homeostatic proliferation of T-cell clones that differ from the clonotypes involved in these immune responses. Accordingly, a decreased alloantigen-induced *ex vivo* response was observed for the T cells of irradiated mice regardless of an adoptive transfer of the spleen cells of non-immunized or immunized mice (*Fig. 2B*).

Lymphopenia could drive the homeostatic proliferation of potentially autoreactive clones. Of particular note, virtually all donor CD8^+^CD44^+^ T cells in our study expressed CD5. Several studies have indicated that the level of CD5 expression could correlate with the avidity of a T-cell receptor (TCR) to self- MHC-peptide complexes [22, 23, 24]. Interaction with self-MHC is required for T cells to proliferate under lymphopenic conditions [25, 26], and T lymphocytes with the highest level of CD5 expression (i.e., naive T cells) have the greatest homeostatic proliferation potential [3]. Accordingly, naive T cells could be the main source of virtual memory cells in the lymphopenic host [15, 26]. Consistent with these findings, we observed a 1.5-fold increase in the relative cell count of donor CD8^+^CD44^+^CD62L^+^CD5^+^ T lymphocytes compared to all controls (*Fig. 5A,B*).

Naive T cells are very radiosensitive [28], and total body irradiation can diminish the population of these cells (*Fig. 4D*). Therefore, we assume that under lymphopenia, without competition for self-MHC-peptide complexes, adoptively transferred donor naive T cells can rapidly acknowledge tonic signals for proliferation [29, 30] and acquire the phenotype of central memory cells (*Fig. 4D*). Thus, it seems possible that, in the lymphopenic host, the memory phenotype of T cells was a consequence of the interaction between TCR and MHC/peptide complexes and homeostatic proliferation, rather than indicative of the actual antigenic experience of this T cell. We have recently shown that in mice transgenic for the β-chain TCR, T cells expressing transgenic TCRβ predominantly show the phenotype of naive cells because of the significant competition for self-MHC-peptide complexes; T cells with endogenous TCRβ express the phenotype of effectors and memory cells as a consequence of the excessive amount of ligands available for recognition [31].

Intriguingly, in the lymphopenic host, donor CD8^+^ T cells acquire a phenotype strikingly different from that of recipient CD8^+^ T cells. CD8^+^ cells comprise 70% of donor CD3^+^ lymphocytes and predominantly carry the phenotype of the central memory cells CD44^+^CD62L^+^. Furthermore, virtually all donor CD8^+^ lymphocytes have the CD49dhi phenotype and express CD122; the expression level of these markers in the subset of donor CD44^+^CD62L^+^ cells is significantly higher compared to that for the respective subpopulation of recipient CD8^+^ T cells.

Thus, we have demonstrated that the population of donor CD8^+^ T cells formed under homeostatic proliferation in the irradiated host acquires the CD44^+^CD62L^+^CD122^+^CD49dhi phenotype, combining some phenotypic characteristics of true memory cells (CD44^+^CD62L^+^CD49dhi) and those of suppressive CD8^+^ T cells (CD44^+^CD62L^+^CD122^+^) [18]. Furthermore, these donor T cells express CXCR3, another marker of suppressive CD8^+^CD122^+^ cells [17]. Considering these findings, we speculate that the adoptive transfer of syngeneic lymphocytes to an irradiated host can lead to the formation of a unique CD8^+^ T-cell subset of donor cells exhibiting suppressive activity.

## CONCLUSIONS

Consistent with previous studies, our experimental data further prove that expression of CD44 on T cells does not always indicate the actual antigenic experience of a T cell and does not necessarily lead to the acquisition of the functional properties of true memory T cells. This means that identification of CD8^+^ memory T cells based solely on their surface phenotype is incorrect and requires confirmation through functional tests. In this study, CD8^+^ T lymphocytes adoptively transferred to the irradiated lymphopenic host were converted to T_ML_ cells that shared the phenotypic features of true memory cells and suppressive CD8^+^ T lymphocytes. This was accompanied through a significant deterioration of the functional state of the recipient’s immune system, whose T cells poorly responded to the alloantigens and bacterial antigens. Memory-like CD8^+^ T cells [32] and suppressive CD8^+^CD44^+^CXCR3^+^ T cells [17] are likely to exist in the human organism. Thus, the adoptive transfer aimed at restoring the count of immune cells in peripheral organs can lead to clinically unfavorable outcomes: i.e., a weaker response to antigens and, hence, increased predisposition or vulnerability to infectious diseases.

## Abbreviations

MHC: major histocompatibility complex
TML: memory-like T cell
MLR: mixed lymphocyte reaction
cpm: counts per minute
AT: adoptive transfer
TCR: T-cell receptor
